# Low frequency pulsed electromagnetic fields exposure alleviate the abnormal subchondral bone remodeling at the early stage of temporomandibular joint osteoarthritis

**DOI:** 10.1186/s12891-022-05916-3

**Published:** 2022-11-16

**Authors:** Yuanjun Ma, Xiaohua Chen, Feng He, Shi Li, Rui He, Qian Liu, Qingshan Dong, Shuncheng Zhou, Hui Miao, Qian Lu, Feifei Li, Hongxu Yang, Mian Zhang, Yuan Lin, Shibin Yu

**Affiliations:** 1 State Key Laboratory of Military Stomatology, National Clinical Research Center for Oral Diseases, Shaanxi Key Laboratory of Oral Diseases, School of Stomatology, the Fourth Military Medical University, Xi’an, Shaanxi 710032 People’s Republic of China; 2 Department of Stomatology, Chinese PLA General Hospital of Central Theater Command, Wuhan, 430070 People’s Republic of China; 3 Department of Stomatology, Seventh Medical Center of Chinese PLA General Hospital, Beijing, 100700 People’s Republic of China

**Keywords:** Temporomandibular joint, Osteoarthritis, Subchondral bone, Pulsed electromagnetic field, Osteogenesis

## Abstract

**Background:**

Temporomandibular joint osteoarthritis (TMJOA) is characterized by abnormal subchondral bone remodeling and cartilage degeneration. As a non-invasive biophysical technology, pulsed electromagnetic field (PEMF) treatment has been proven to be efficient in promoting osteogenesis. However, the potential bone protective effect and mechanism of PEMF on abnormal subchondral bone remodeling in TMJOA are unknown.

**Methods:**

Unilateral anterior crossbite (UAC) was used to create TMJOA model in rats, and 17β-estradiol (E_2_) were injected daily to mimic patients with high-physiological levels of estrogen. Mouse osteoblast-like MC3T3-E1 cells treated with recombinant murine IL-1β was used to establish inflammatory environment in vitro. The treatment group were subjected to PEMF (2.0mT, 15 Hz, 2 h/d). Micro-CT scanning, histological staining, real-time PCR and western blotting assays were preformed to observe the changes in the subchondral bone.

**Results:**

Abnormal resorption of subchondral bone induced by UAC, characterized by decreased bone mineral density, increased osteoclast activity and expression of osteoclast-related factors (RANKL) and down-regulated expression of osteogenesis-related factors (OPG, ALP, Runx2 and OCN) at the early stage, could be reversed by PEMF exposure, which was similar to the effect of estrogen. In addition, PEMF exposure and E_2_ supplement may have a synergistic effect to some extent. Moreover, PEMF exposure could promote the ALP activity and osteogenic mineralization ability of MC3T3-E1 cells. PEMF promoted the expression of factors related to Wnt/β-Catenin signal pathway both in vivo and in vitro.

**Conclusions:**

Appropriate PEMF exposure have a protective effect on subchondral bone in TMJOA at early stage, in which canonical Wnt/β-Catenin pathway may be involved. PEMF may be a promising biophysical approach for early intervention of TMJOA in clinic.

**Supplementary Information:**

The online version contains supplementary material available at 10.1186/s12891-022-05916-3.

## Background

Temporomandibular disorders (TMD) are defined as a subgroup of craniofacial pain problems that involve the temporomandibular joint (TMJ), masticatory muscles, and associated head and neck musculoskeletal structures. Patients with TMD most frequently present with pain, limited or asymmetric mandibular motion, and TMJ sounds [1]. The prevalence of TMD is reported as 20 to 40%, and the ratio of female-to-male seeking TMD treatment is as high as 3:1 to 9:1 [2]. The cause of TMD is now considered multifactorial, with biologic, behavioral, environmental, social, emotional and cognitive factors, alone or in combination, contributing to the development of signs and symptoms of TMD [1, 3, 4]. Temporomandibular joint osteoarthritis (TMJOA) is the most severe manifestation of TMD. Its typical pathological features include abnormal subchondral bone remodeling and cartilage degeneration [5]. Reduced bone mineral density and increased subchondral bone remodeling have been observed in the early stages of osteoarthritis (OA). Abnormal subchondral bone remodeling plays an important role in the pathogenesis of OA [6]. Abnormal subchondral bone changes could lead to a less compliant trabecular bone, which thereby transfers excessive mechanical stress to the overlying articular cartilage, resulting in cartilage degeneration [7, 8]. In addition, the clinical diagnosis of OA is mainly based on the radiographic changes of subchondral bone [9]. Therefore, reversing the early subchondral bone resorption may be the key to the treatment of TMJOA.

As a specific joint, TMJ is different from other large joints of limbs in anatomy. Firstly, the surface of limb joints is covered with hyaline cartilage, while the surface of the TMJ condyle is covered with fibrocartilage. Secondly, the cartilage and subchondral bone of limb joints are separated by a physiological barrier composed of tidal line and calcified matrix, so the communication between them is limited. However, the trabecular bone of the subchondral bone extends directly into the condylar cartilage in TMJ. Thirdly, unlike OA in limb joints have greater female predilection that occur postmenopausally, a large proportion of women with TMD are between 18 and 45 years of age [10]. Therefore, there may be differences in the biological effect and molecular mechanism of OA between TMJ and large joints of limbs.

As a safe and non-invasive biophysical method, pulsed electromagnetic field (PEMF) stimulation has been widely used in the treatment of skeletal diseases since approved by the food and drug administration (FDA) in 1979 [11]. A number of studies have confirmed the satisfactory effect of PEMF in the treatment of fresh fractures, delayed fractures, ununited fractures and OA of large joints of limbs [12–14]. More recently, growing evidence had shown that PEMF has positive effects on maintaining bone mass and strength in rodent osteoporosis models induced by estrogen deficiency, disuse and diabetes [15–17]. Some studies in vitro also had shown that PEMF have the ability to promote osteoblast differentiation and mineralization [18, 19]. Although PEMF has been successfully used in many bone diseases, its potential mechanism is not fully understood. Some studies demonstrated that the classical Wnt/β-Catenin signal pathway plays an important role in regulating bone development and formation [20]. In addition, classical Wnt/β-Catenin signal pathway has been found in the osteogenic physiological activity of PEMF in vivo and in vitro [21, 22]. Therefore, classical Wnt/β-Catenin signal pathway may also be involved in the effect of PEMF on subchondral bone remodeling in TMJOA.

In this study, for the first time, we investigated the effect of PEMF on subchondral bone degeneration at the early stage of TMJOA and explored the possible molecular mechanism, so as to provide the possibility for early intervention of TMJOA.

## Materials and methods

### Experimental animals and groups

**Fig. 1:**
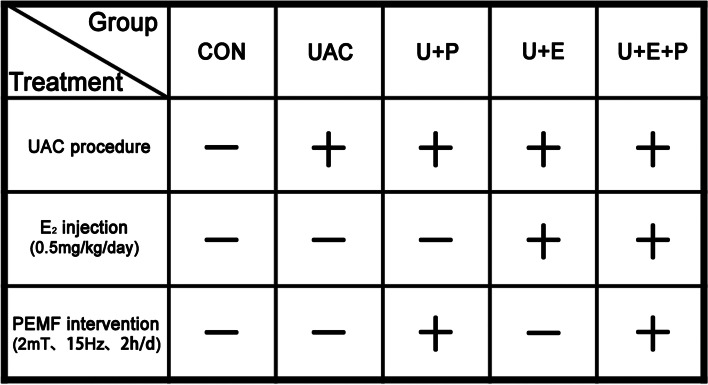
The animals grouping

One hundred and twenty 6-week-old female Sprague-Dawley (SD) rats, weighing 170–190 g, were provided by the Animal Center of the Fourth Military Medical University. All procedures have been examined and approved by the Institutional Ethics Committee of School of Stomatology, the Fourth Military Medical University (2021–080). According to the experimental time point (3 and 6 weeks), the rats were randomly assigned to 2 groups, and each group was divided into 5 subgroups: control group (CON), unilateral anterior crossbite group (UAC), unilateral anterior crossbite with PEMF intervention group (U + P), unilateral anterior crossbite with estrogen injection at 0.5 mg/kg/d group (U + E) and unilateral anterior crossbite combined with estrogen injection at 0.5 mg/kg/d and PEMF group (U + E + P), with 12 rats in each subgroup. During the study, all rats were placed in a pathogen-free room and fed sterilized food and redistilled water. The group design is shown in Fig. 1.

The UAC model was established in the rats as previously described. Briefly, under deep anesthesia with 1% pentobarbital sodium (0.35 ml/100 g weight), a small metal tube (length = 2.5 mm, inside diameter = 3 mm) was bonded to the left maxillary incisor with zinc phosphate cement, and another metal tube (length = 4.5 mm, inside diameter = 2.5 mm) was bonded to the left mandibular incisor of the rats. The end of latter tube was bent to a 135° angle to guide the mandible forward to create a UAC relation of the left side incisors. The rats in the CON group were anesthetized with the same method, but the tubes were not retained on the incisors. Each operation was completed within 3 minutes and all efforts were made to minimise suffering. During the entire experimental period, no detachment of the metal tube was found and the rat eating is not affected. From 1 week before the UAC operation, the rats in U + E and U + E + P groups were injected with 17β-estradiol (E_2_, ab120657, Abcam, Cambridge, MA, UK) subcutaneously daily in the morning, at a dose of 0.5 mg/kg/day until the end of the experiment. E_2_ was dissolved in dimethyl sulfoxide (DMSO) and diluted to 0.2 mg/ml in saline immediately before administration. The other groups received saline injections. From the second day of UAC operation, the rats in U + P and U + E + P group were irradiated for 2 hours every afternoon until the end of the experiment, in which the magnetic field parameters were constant. The rats in the other groups were put in the same device for the same time, but the magnetic field intensity was 0 mT.

**Fig. 2:**
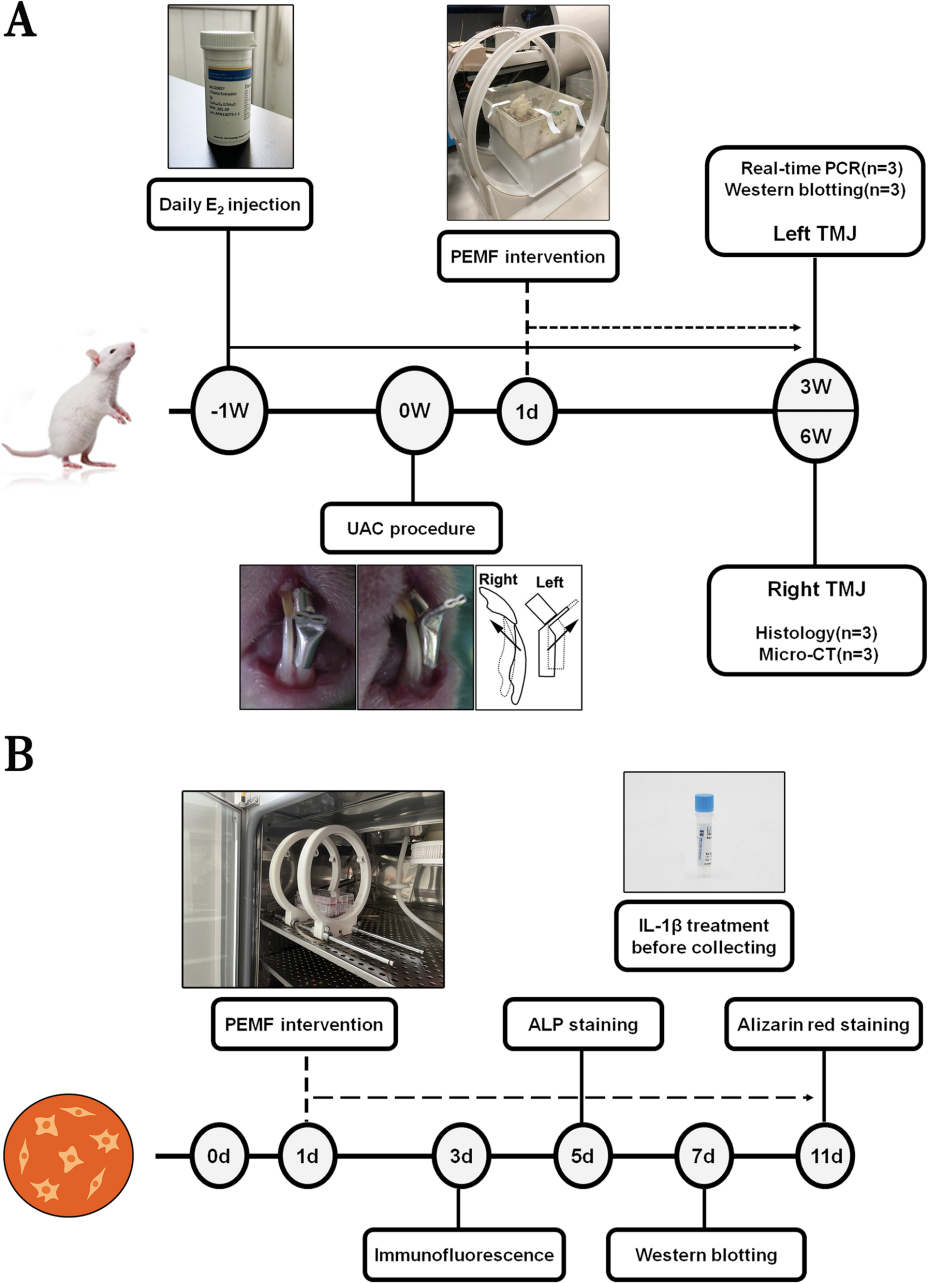
The timeline and procedure of the entire experiment

The body weight of rats was measured at the predetermined time point (3 and 6 postoperative weeks). Then, experimental rats were sacrificed with a single intraperitoneal injection of overdose pentobarbital sodium. The left TMJs from 12 rats were used for real-time PCR (*n* = 6) and western boltting assays (*n* = 6). The right temporomandibular joints were randomly divided into two groups, which were used for paraffin section staining (*n* = 6) and micro-CT scanning (*n* = 6) respectively. Our previous studies have demonstrated that there is no significant difference in OA pathological findings between left and right TMJ subchondral bone induced by UAC [23]. The detailed experimental schedule is shown in Fig. 2A.

### PEMF stimulation and tissue preparation

Rats were exposed to whole-body PEMF stimulation generated by a custom-designed electromagnetic exposure device (GHY-III, FMMU, Xi’an, China). The device consists of a signal generator and three parallel Helmholtz coils (Fig. 1B). The PEMF waveform used in this experiment consisted of a pulsed burst (burst width, 5 msec; pulse width, 0.2 msec; pulse wait, 0.02 msec; burst wait, 60 msec; pulse rise, 0.3 msec; pulse fall, 2.0 msec) at a frequency of 15 Hz. The intensity of the magnetic fields was accurately measured using a Gaussian meter (455DMP gauss meter, Lake Shore Cryogenic Company, Westville, Ohio), and the peak intensity of the magnetic fields was approximately 2.0 mT. Previous studies have confirmed that this special PEMF waveform can promote osteoblasts function and osteogenesis [16, 17].

Six right intact TMJs of rats in each group were dissected and fixed in 4% paraformaldehyde (pH 7.4) at 4 °C for 24 hours. Three of them were randomly selected and immediately sent to micro-CT scan, and the other three were decalcified in 10% ethylene diamine tetraacetic acid (EDTA) for 2 months, followed by being embedded in paraffin wax and cut into 5 μm-thick serial mid-sagittal sections.

### Micro-CT analysis

TMJs were scanned in vitro using a micro-CT system (Inveon, Siemens, MUC, Bavaria, Germany) to examine the ultrastructural changes of the subchondral bone, as previously reported [24]. The detected ultra-parameters included BV/TV (the ratio of bone volume to tissue volume), BS/BV (the ratio of bone surface area to bone volume), Tb. Th (trabecular thickness), Tb. Sp (trabecular separation), Tb. N (trabecular number). The means of every three interested regions were used for the statistical analysis.

### Hematoxylin-eosin and TRAP staining

To observe the changes of morphology and osteoclasts in subchondral bone, the central sagittal paraffin sections of each tissue were performed by HE and tartrate resistant acid phosphatase (TRAP) staining, following the manufacturer’s program (Sigma,387-A, St. Louis, MO, USA) and our previous reports [24]. The morphology of subchondral bone was observed under Leica optical microscope (Leica 2500, Hesse, Germany). The double-blind method was used, and the high magnification visual field in each section was randomly selected to count the TRAP positive cells (Oc. N, TRAP-positive cell with 3 or more nuclei represents osteoclast). The average number of osteoclasts in 5 visual fields was statistically analyzed.

### Real-time PCR and western blotting analysis

Under stereoscopic microscope, the subchondral bone of the condyle was dissected carefully, and the subchondral bone at the 2 mm below the cartilage-bone junction was cross-sectioned. All subchondral bones were preserved at − 80 °C. The subchondral bone samples from every subgroup were homogenised, and the total RNA was extracted using the Tripure Isolation Reagent (Roche Life Science, US). All genes were analysed using CFX 96 real-time PCR (Bio-Rad, Hercules, California, USA). The primer sequences were designed and synthesized based on the mRNA sequences obtained from the NCBI database, as shown in Table 1. Each target gene was analyzed three times relative to β-actin, and the mean values were calculated using the 2^−ΔΔCt^ method.

**Table 1:** Gene primers

Gene	Froward	Reverse
β-Catenin	ACAAGCCACAGGACTACAAGAAACG	TCAGCAGTCTCATTCCAAGCCATTG
Wnt1	GGGTTTCTGCTACGTTGCTACTGG	AGGTTCGTGGAGGAGGCTATGTTC
LRP5	ACACCAACATGATTGAGTCCTCCAAC	AGGTTCCAGTCGGTCCAGTAGATG
ALP	AGGAGGGAGTCGTTCATGGAGTG	ACCAGTGGCAGCAGCAATGTTC
COL1A1	TGTTGGTCCTGCTGGCAAGAATG	GTCACCTTGTTCGCCTGTCTCAC
BMP2	ACCCTTTGTATGTGGACTTCAGTGATG	CTATGGCATGGTTGGTGGAGTTCAG
OCN	GGACCCTCTCTCTGCTCACTCTG	ACCTTACTGCCCTCCTGCTTGG
Runx2	CTTCGTCAGCGTCCTATCAGTTCC	TCCATCAGCGTCAACACCATCATTC
RANKL	GAGCGAAGACACAGAAGCACTACC	GAGCCACGAACCTTCCATCATAGC
OPG	CCAGACGAGATTGAGAGAACGAGAAAG	AAGTGCTTGAGTGCGTACATCAGG
β-actin	AAGTGCTTGAGTGCGTACATCAGG	ACTGTGTTGGCATAGAGGTCTTTACG

The total protein of each group in vivo and in vitro was extracted by Tripure Isolation Reagent (Roche), and the protein concentration was detected by BCA Protein Assay kit. The protein extracts were diluted to 4:1 with 5 × loading buffer. Equal amounts of proteins from each group were separated by 10% sodium dodecyl sulfate-polyacrylamide gel electrophoresis, and transferred to polyvinylidene fluoride (PVDF) membranes. The membranes were blocked in BSA for 1 h, then respectively incubated overnight with primary antibodies to OPG (Affinity Biosciences, DF6824), RANKL (Affinity Biosciences, AF0313), β-Catenin (Affinity Biosciences, AF6266), GSK3β (Affinity Biosciences, AF5016), p-GSK3β (Affinity Biosciences, AF2016), COL-I (Abcam, ab270993), OCN (Affinity Biosciences, DF12303) and β-Tubulin (Affinity Biosciences, AF7011) at 4 °C. Membranes were then incubated with a 1:3000 dilution of HRP-conjugated goat anti-rabbit antibody for 1 h at RT. The SuperSignal West Pico chemiluminescent substrate kit (Thermo Scientific, Rockford, IL) was used to visualize the blots according to the manufacturer’s instructions. Then, the membranes were scanned by the Chemi-Doc XRS + WB luminous imaging system. Image Lab 5.2.1 software was used for analysis after image acquisition.

### Cell culture

The mouse osteoblast-like MC3T3-E1 cells (ATCC, Manassas, VA) were cultured in α-MEM medium containing 100 units/ml penicillin, 100 mg/ml streptomycin and 10% FBS with a water-saturated atmosphere of 5% CO_2_ at 37 °C. Cells were treated with recombinant murine IL-1β (PeproTech, USA) in 20 ng/ml for 24 h to simulate the inflammatory condition before collection. Cell suspension was seeded into a 6-well cell culture plate (1 ml/ well) with a culture medium density of 1 × 10^5^ cells/ml. The growth medium was replaced with osteoinductive medium composed of a-MEM containing 10% fetal bovine serum, 1% penicillin-streptomycin, 50 mg/ml ascorbic acid and 4 mM β-glycerophosphate, then the cells were exposed to PEMF stimulation for 2 h/d. To investigate the changes of ALP in cells, ALP staining was performed post 5-day PEMF stimulation. To investigate the expression of osteogenesis-related proteins, western blotting analysis was performed post 7-day PEMF stimulation. To investigate the osteogenic mineralization in cells, alizarin red staining was performed post 11-day PEMF stimulation. Cell suspension was seeded into a 24-well cell culture plate (0.5 ml/ well), and the density of the culture medium was 1.5 × 10^4^ cells/ml. Cells were exposed to PEMF stimulation for 2 h/d after adhesion. To investigate the changes of cell morphology and the expression of β-Catenin, morphological collection and immunofluorescence staining were performed post 3-day PEMF stimulation. The detailed experimental schedule is shown in Fig. 2B.

### Immunofluorescence

MC3T3-E1 cells were stained with immunofluorescence to evaluate the expression of β-Catenin. Cells were incubated with primary antibody to β-Catenin (Affinity Biosciences, AF6266) on round, sterilized glass cover slips (14 mm), then incubated with Alexa647-labeled secondary antibody diluted by DAPI for 2 h. Cover slip were placed on clean slides and sealed with anti-fluorescence quenching sealing liquid and stored at 4 °C, then observed by confocal laser scanning microscope (FV1000, Olympus, Tokyo, Japan).

### ALP and alizarin red staining

After 5 and 9 days of PEMF stimulation, ALP and alizarin red staining were performed respectively. Cells were fixed with 4% paraformaldehyde solution, then BCIP/NBTALP color development kit was used for ALP staining [25]. Alizarin red staining was performed as previously reported [26]. The image was observed under a Leica optical microscope.

### Statistical analysis

The SPSS 21.0 package (SPSS Inc., Chicago, IL, USA) was used to analyse and describe the data. All data collection and analysis were completed blindly. For comparisons of the means of measurement among the 5 groups, one-way ANOVA test was applied, and Tukey’s multiple comparisons test was used to compare between every 2 groups. All values were presented as the mean, with 95% confidence intervals (95% CI), and *P* values of < 0.05 were defined as being statistically significant.

## Results

### PEMF reversed the early effect of E_2_ supplement on the body weight

**Fig. 3:**
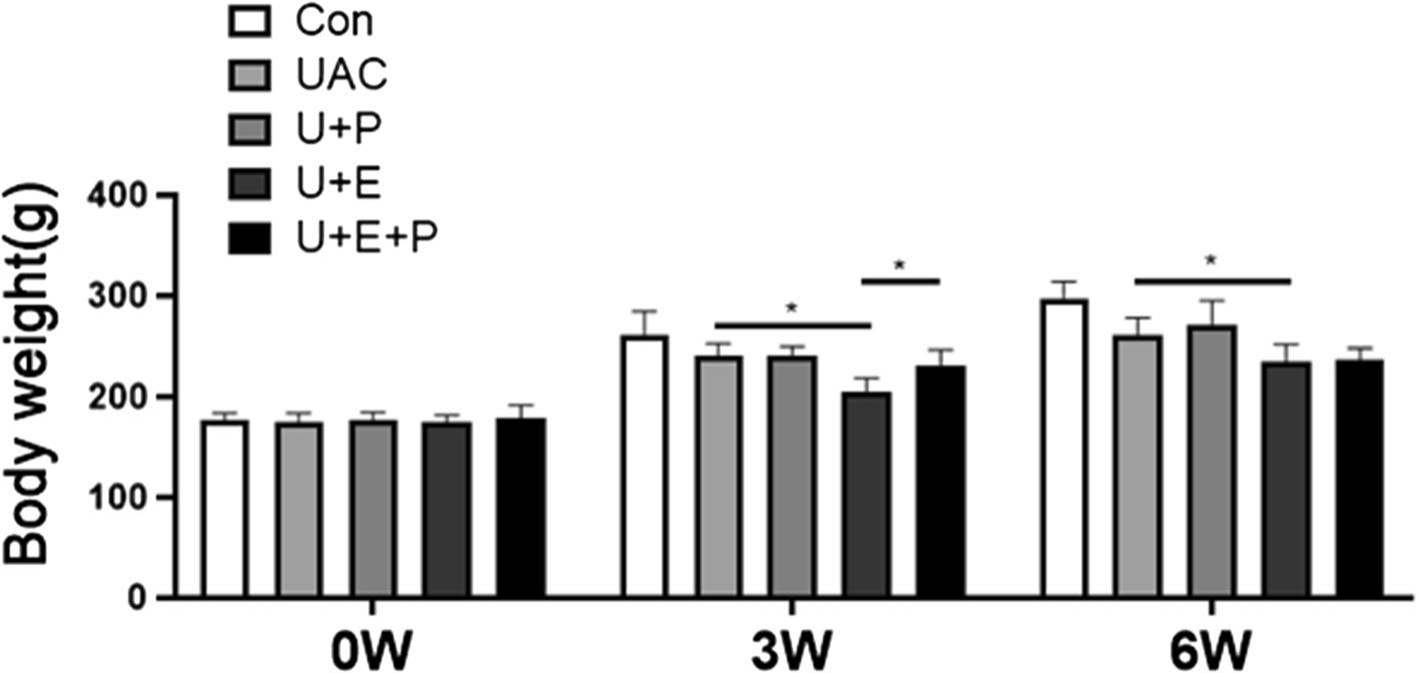
Body weights of the different groups (*n* = 12). *: *P* < 0.05

As shown in Fig. 3, no significant difference was found in body weight among CON, UAC and U + P groups at 3 W and 6 W (*P* > 0.05). However, E_2_ supplement significantly reduced the body weight of rats at 3 W and 6 W (*P* < 0.05, UAC vs U + E), which was consistent with previous study [27]. PEMF intervention significantly reversed the weight loss of rats with E_2_ supplement at 3 W (*P* < 0.05, U + E vs U + E + P), but not at 6 W. These results suggest that PEMF intervention may function on the whole body of experimental animals.

### PEMF reversed abnormal bone resorption in subchondral bone induced by UAC

**Fig. 4:**
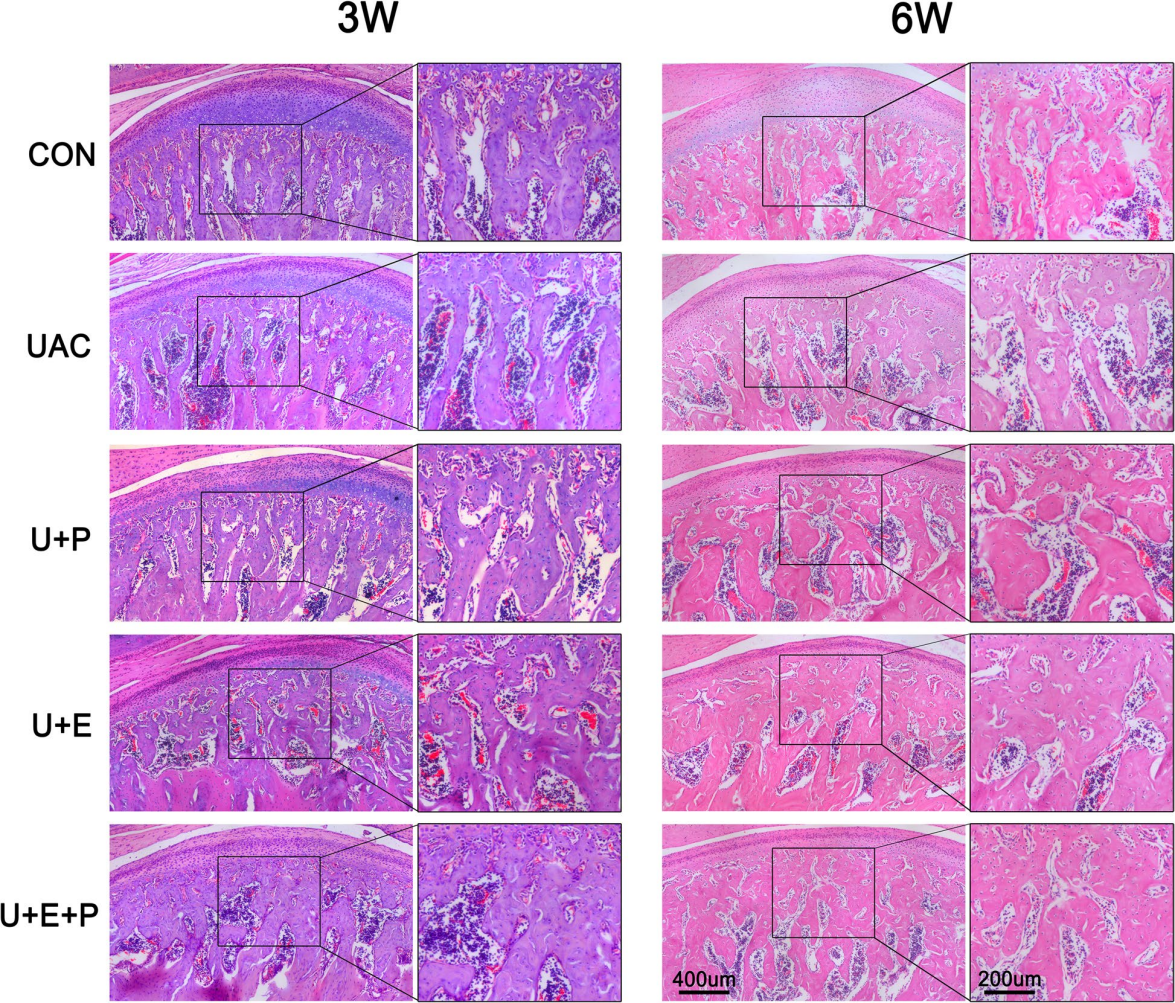
The morphology of condylar subchondral bone of the different groups

**Fig. 5:**
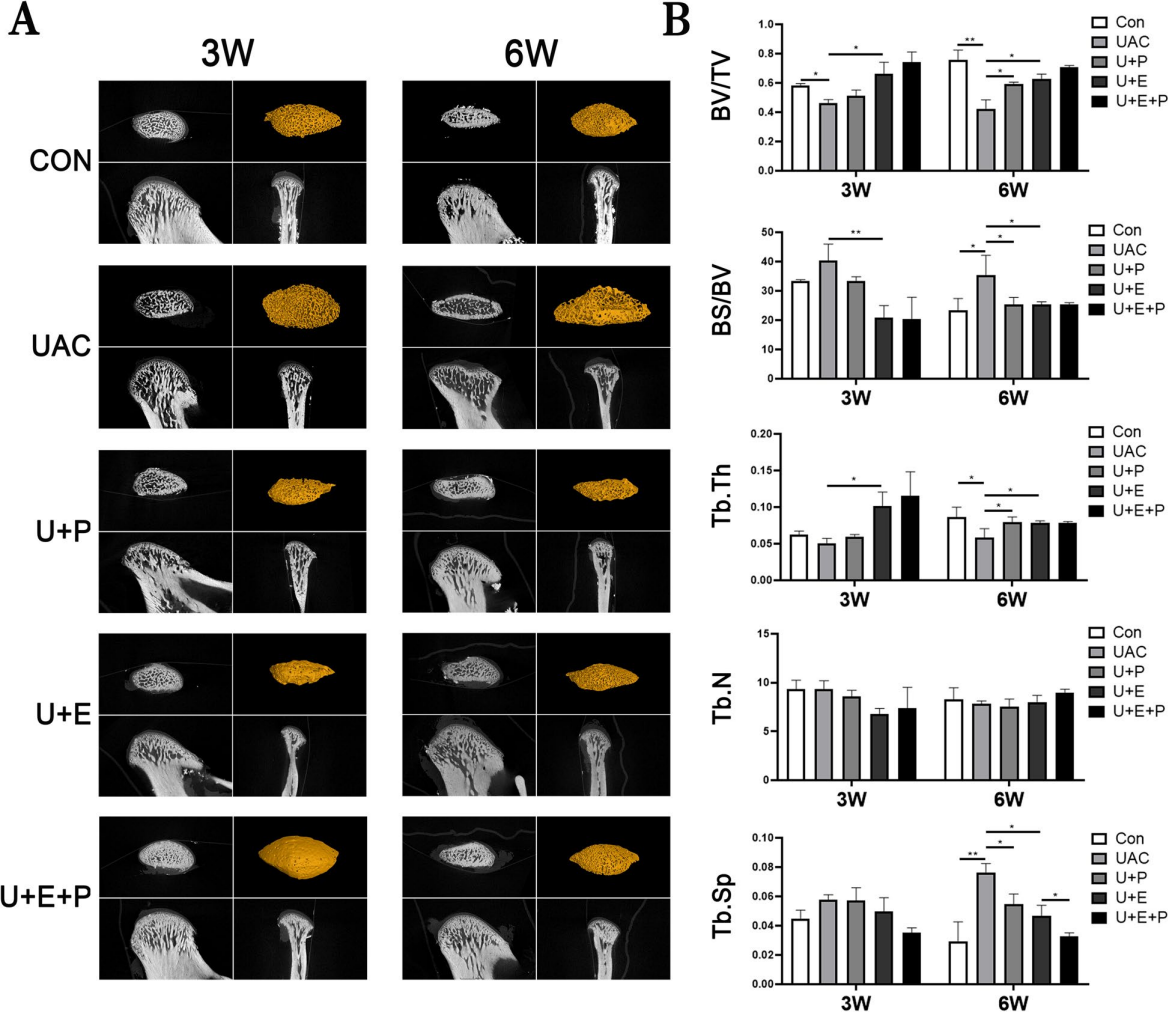
**A ** Representative images of condylar subchondral bone through micro-CT scanning in the sagittal section view and a 3D reconstructed view. **B** Comparisons of the bone ultra-parameters among different groups (*n* = 6). *: *P* < 0.05, **: *P* < 0.01

As shown in Figs. 4 and 5A, diffuse subchondral bone loss, enlargement of bone marrow cavity, rough and uneven surface of subchondral bone were observed in UAC group at 3 W and 6 W. Compared with CON group, the BV/TV (3 W: *P* < 0.05; 6 W: *P* < 0.01) and Tb. Th (6 W: *P* < 0.05) decreased significantly, while BS/BV (6 W: *P* < 0.05) and Tb. Sp (6 W: *P* < 0.01) increased significantly in UAC group (Fig. 5B).

PEMF intervention reversed the loss of subchondral bone and the enlargement of bone marrow cavity and reduced the surface roughness of subchondral bone induced by UAC in 3 W and 6 W U + P group (Figs. 4 and 5A). Compared with UAC group, the BV/TV (6 W: *P* < 0.05) and Tb. Th (6 W: *P* < 0.05) increased significantly, while BS/BV (6 W: *P* < 0.05) and Tb. Sp (6 W: *P* < 0.05) decreased significantly in U + P group (Fig. 5B).

After E_2_ supplement, the loss of subchondral bone and the enlargement of bone marrow cavity were also inhibited. Compared with UAC group, the BV/TV (3 W: *P* < 0.05; 6 W: *P* < 0.05) and Tb. Th (3 W: *P* < 0.05; 6 W: *P* < 0.05) were significantly increased, while the BS/BV (3 W: *P* < 0.01; 6 W: *P* < 0.05) and Tb. Sp (6 W: *P* < 0.05) decreased significantly in U + E group, which was consistent with previous study [28]. Moreover, combined with PEMF intervention and E_2_ supplement, the effect of subchondral bone loss and enlargement of bone marrow cavity was further alleviated, as shown by the further decrease of Tb. Sp (6 W: *P* < 0.05, U + E + P vs U + E) (Fig. 5B).

### PEMF reduced osteoclast activity induced by UAC

**Fig. 6:**
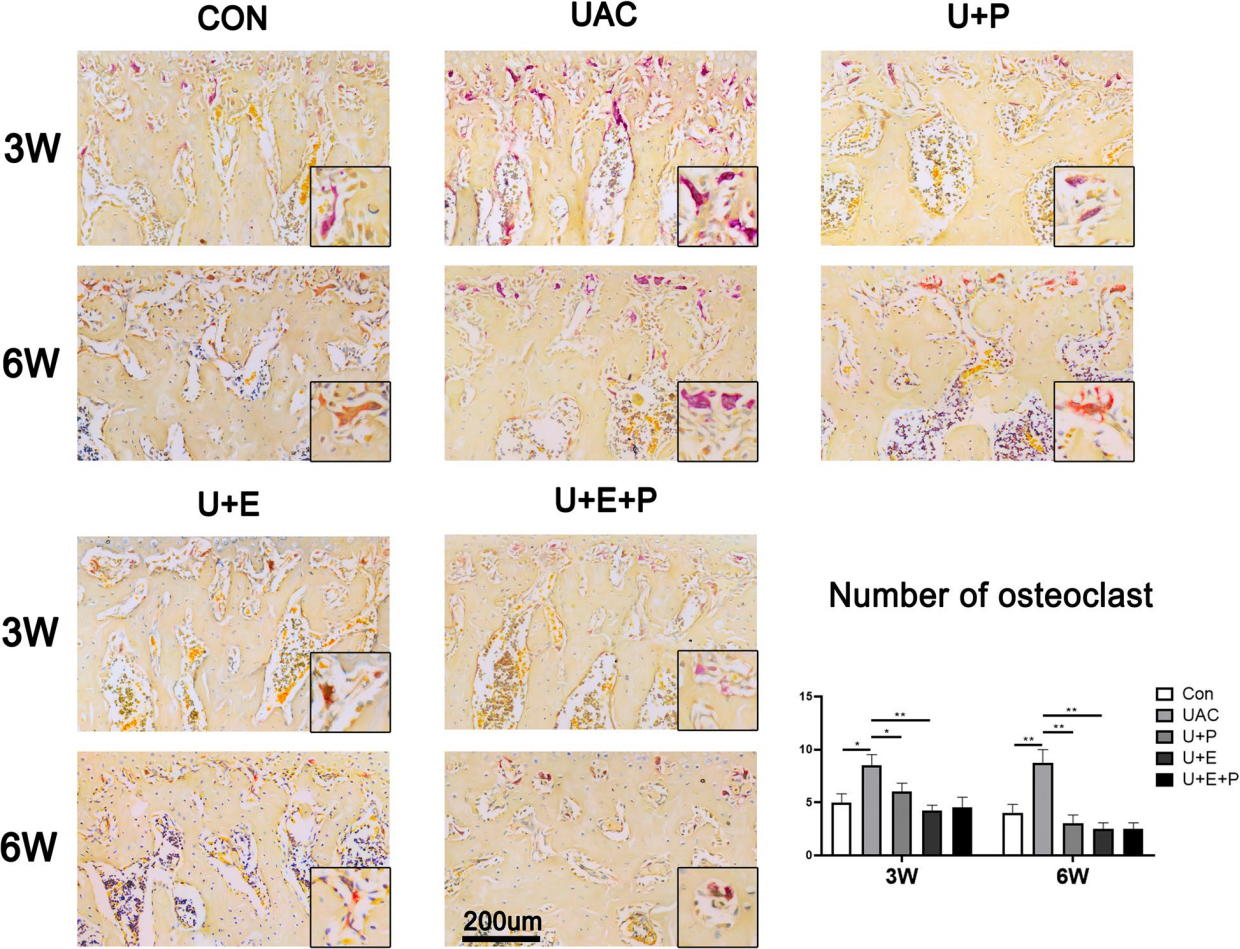
TRAP staining of condylar subchondral bone among different groups and comparisons of the number of osteoclasts among different groups (*n* = 6). *: *P* < 0.05, **: *P* < 0.01

As shown in Fig. 6, the number of TRAP positive cells increased significantly in UAC group compared with that in CON group at 3 W and 6 W (3 W: *P* < 0.05; 6 W: *P* < 0.01). PEMF intervention (3 W: *P* < 0.05, U + P vs UAC; 6 W: *P* < 0.01, U + P vs UAC) and E_2_ supplement (3 W: *P* < 0.01, U + E vs UAC; 6 W: *P* < 0.01, U + E vs UAC) significantly decreased the number of TRAP-positive cells. However, there was no significant difference in the number of TRAP-positive cells between the U + E + P group and U + E group (*P* > 0.05).

### PEMF reversed the decreased expression of osteogenesis-related factors and the increased expression of osteoclast-related factors induced by UAC

**Fig. 7:**
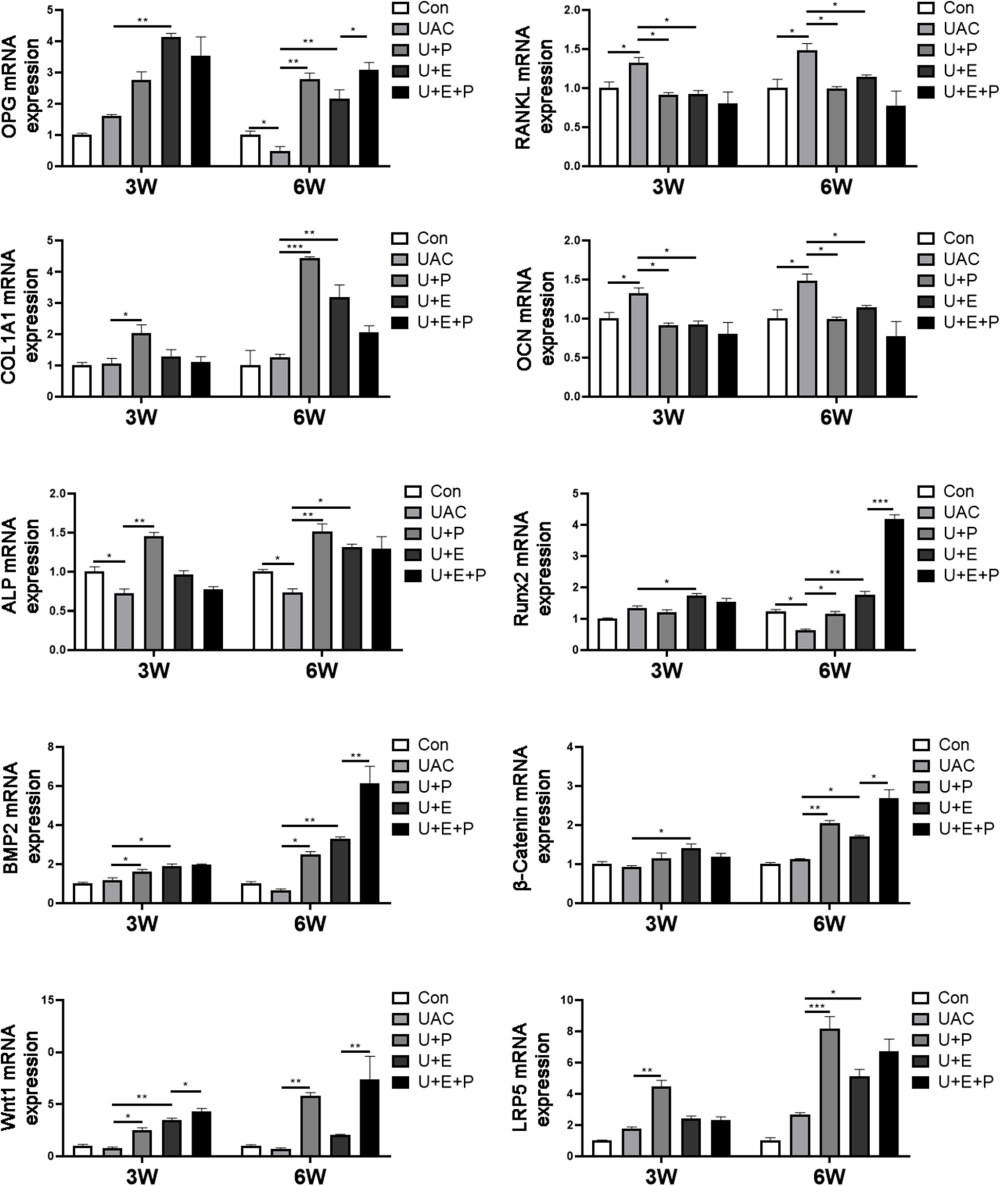
The mRNA expression of osteogenesis-related factors (*n* = 6). *: *P* < 0.05, **: *P* < 0.01, ***: *P* < 0.001

As shown in Fig. 7, compared with CON group, the mRNA expression of OPG (6 W: *P* < 0.05), ALP (6 W: *P* < 0.001), Runx2 (6 W: *P* < 0.05) and OCN (3 W: *P* < 0.05; 6 W: *P* < 0.05) decreased while the mRNA expression of RANKL (3 W: *P* < 0.05; 6 W: *P* < 0.05) increased significantly in UAC group at 3 W and 6 W. PEMF intervention significantly increased the mRNA expression of OPG (6 W: *P* < 0.01), COL1A1 (3 W: *P* < 0.05; 6 W: *P* < 0.001), OCN (3 W: *P* < 0.01; 6 W: *P* < 0.01), BMP2 (3 W: *P* < 0.05; 6 W: *P* < 0.05) and Runx2 (6 W: *P* < 0.05), and decreased the mRNA expression of RANKL (3 W: *P* < 0.05; 6 W: *P* < 0.05) in U + P group compared with that in UAC group.

In addition, E_2_ supplement could also reverse the down-regulated mRNA expression of OPG (3 W: *P* < 0.01; 6 W: *P* < 0.01), COL1A1 (6 W: *P* < 0.01), OCN (6 W: *P* < 0.05), ALP (3 W: *P* < 0.05; 6 W: *P* < 0.01), Runx2 (3 W: *P* < 0.05; 6 W: *P* < 0.01), BMP2 (3 W: *P* < 0.05; 6 W: *P* < 0.01) and the up-regulated mRNA expression of RANKL (3 W: *P* < 0.05; 6 W: *P* < 0.05) in U + E group compared with that in UAC group. After the combined action of PEMF and E_2_, the mRNA expression of OPG (6 W: *P* < 0.05), Runx2 (6 W: *P* < 0.001) and BMP2 (6 W: *P* < 0.01) was further up-regulated in U + E + P group compared with that in U + E group.

**Fig. 8:**
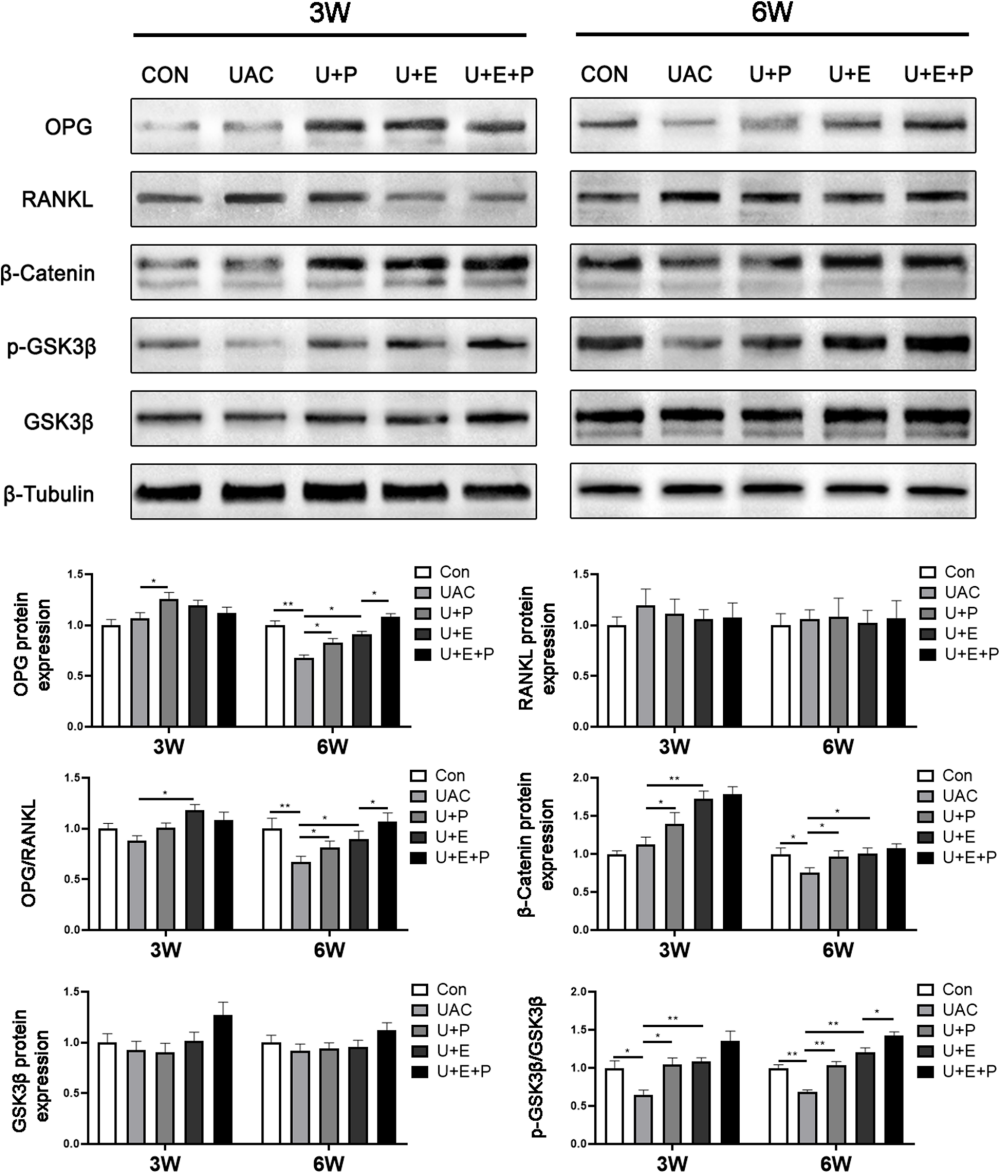
The protein expression of osteogenesis-related factors (*n* = 6). *: *P* < 0.05, **: *P* < 0.01, ***: *P* < 0.001. (All bands come from the same membrane in 3 W and 6 W. The membranes were cut prior to hybridisation with antibodies. After scanned, the membrane is washed in the stripping buffer, then incubated with another antibodies)

Similarly, as shown in Fig. 8, the protein expression of OPG decreased significantly in UAC group (6 W: *P* < 0.01, Con vs UAC), but significantly increased after PEMF intervention (3 W: *P* < 0.05, U + P vs UAC; 6 W: *P* < 0.05, U + P vs UAC) and E_2_ supplement (6 W: *P* < 0.05, U + E vs UAC) respectively. In addition, the combined effect of PEMF and E_2_ significantly increased the protein expression of OPG (6 W: *P* < 0.05, U + E + P vs U + E), but there was no significant difference in RANKL protein expression among the three groups (*P* > 0.05). The ratio of OPG/RANKL was also significantly decreased in UAC group (6 W: *P* < 0.01, Con vs UAC), and increased significantly in U + P group (6 W: *P* < 0.05, U + P vs UAC) and U + E group (3 W: *P* < 0.05, U + E vs UAC; 6 W: *P* < 0.05, U + E vs UAC). Moreover, the ratio of OPG/RANKL was further increased in U + E + P group compared with that in U + E group (6 W: *P* < 0.05).

### PEMF reverses the effect of inflammatory condition on MC3T3-E1 cells induced by IL-1β

**Fig. 9:**
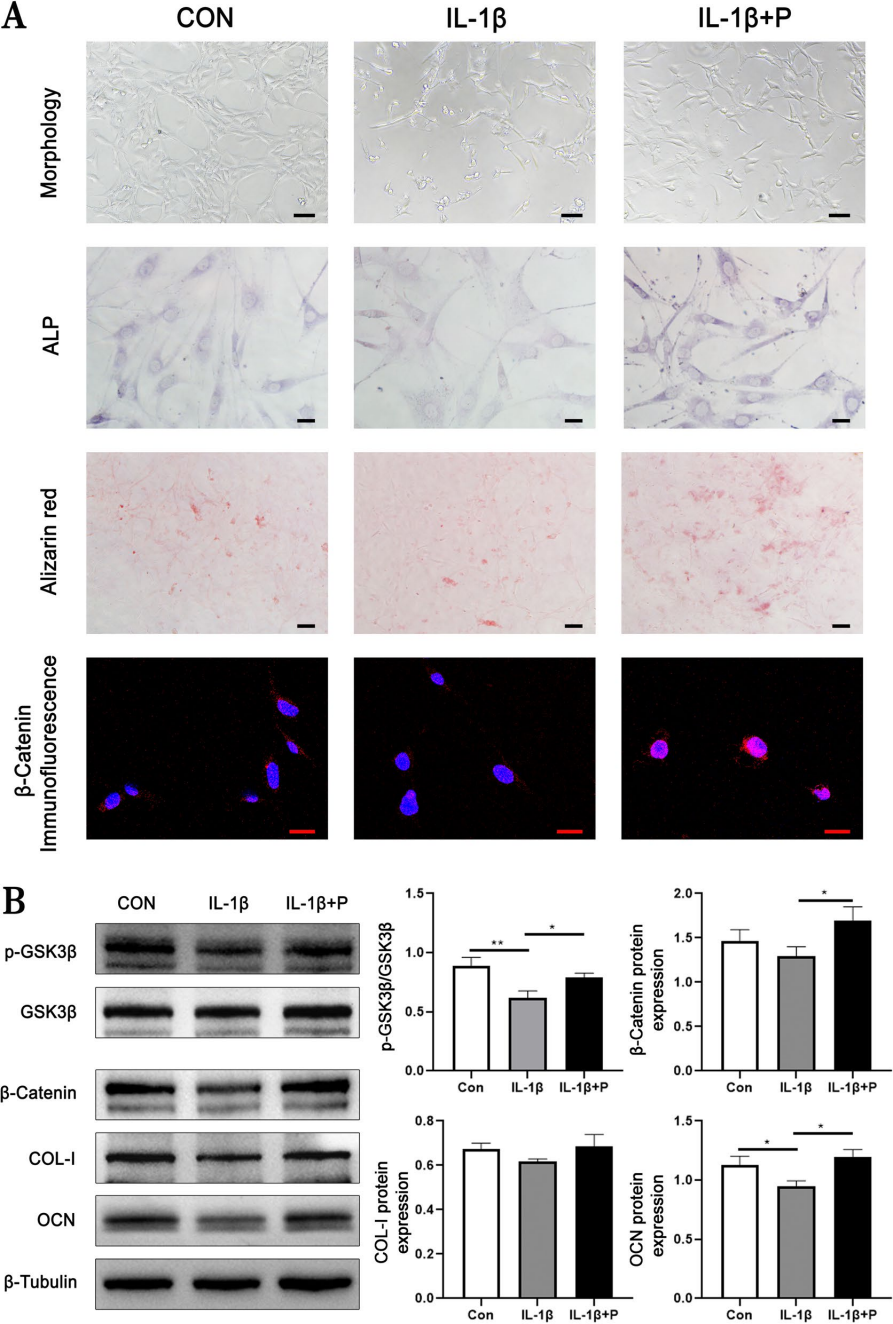
**A** Morphology and staining of MC3T3-E1 cells in each group. Scale bars represent (Morphology:100um, ALP:20um, Alizarin red:100um, Immunofluorescence:20um). **B** The protein expression of osteogenesis-related factors (*n* = 3). *: *P* < 0.05, ***: *P* < 0.001

The morphologies of MC3T3-E1 cells and protein expression of osteogenesis-related factors in each group were shown in Fig. 9A and B. Treated with IL-1β in 20 ng/ml for 24 hours, phenotypic changes and a large number of apoptosis and necrosis in cells were observed. The ALP activity was significantly decreased while the osteogenic mineralization was slightly decreased. In the meantime, the protein expression of OCN was significantly decreased compared with that in CON group (*P* < 0.05).

After the intervention of PEMF, the abnormal morphology in cells was significantly reversed and the number of apoptotic and necrotic cells decreased significantly. The ALP activity, osteogenic mineralization and protein expression of OCN were significantly increased compared with those in IL-1β group (*P* < 0.05). However, there was no significant difference in the protein expression of COL-I among the groups. The above demonstrated that IL-1β treatment induces the significantly decreased of osteogenic ability and osteogenesis-related factors expression, while PEMF reversed the effect of IL-1β and promoted the osteogenic ability of MC3T3-E1 cells.

### PEMF promotes the activation of Wnt/β-catenin signal pathway

As shown in Fig. 7, there was no significant difference in mRNA expression of factors related to Wnt/β-Catenin signaling pathway between UAC group and CON group at 3 W and 6 W. Compared with UAC group, PEMF intervention significantly promoted the mRNA expression of Wnt1 (3 W: *P* < 0.05; 6 W: *P* < 0.01), LRP5 (3 W: *P* < 0.01; 6 W: *P* < 0.001) and β-Catenin (6 W: *P* < 0.01). Meanwhile, the mRNA expression of Wnt1 (3 W: *P* < 0.01), LRP5 (6 W: *P* < 0.05) and β-Catenin (3 W: *P* < 0.05; 6 W: *P* < 0.05) were also promoted by E_2_ supplement. After the combined action of PEMF and E_2_, the mRNA expression of Wnt1 (3 W: *P* < 0.05; 6 W: *P* < 0.01) and β-Catenin (6 W: *P* < 0.05) in U + E + P group was significantly increased compared with that in U + E group.

As shown in Fig. 8, the protein expression of β-Catenin (6 W: *P* < 0.05) and the ratio of p-GSK3β/GSK3β (3 W: *P* < 0.05；6 W: *P* < 0.01) in UAC group was significantly decreased compared with that in CON group at 3 W and 6 W. Compared with UAC group, PEMF intervention significantly increased the protein expression of β-Catenin (3 W: *P* < 0.05; 6 W: *P* < 0.05) and the ratio of p-GSK3β/GSK3β (3 W: *P* < 0.05; 6 W: *P* < 0.01). In the meantime, E2 supplement also significantly increased the protein expression of β-Catenin (3 W: *P* < 0.01; 6 W: *P* < 0.05) and the ratio of p-GSK3β/GSK3β (3 W: *P* < 0.01; 6 W: *P* < 0.01). In addition, the ratio of p-GSK3β/GSK3β in U + E + P group was significantly increased compared with that in U + E + P group at 6 W (*P* < 0.05).

As shown in Fig. 9A, immunofluorescence showed that the expression of β-Catenin in MC3T3-E1 cells treated with IL-1β was significantly decreased compared with that in CON group. PEMF intervention significantly increased the expression of β-Catenin compared with that in IL-1β group. In addition, the expression was mainly located in the nucleus, indicating that the entry of β-Catenin into the nucleus was increased. As shown in Fig. 9B, the ratio of p-GSK3β/GSK3β (*P* < 0.01) in IL-1β group was significantly decreased compared with that in CON group while the ratio of p-GSK3β/GSK3β (*P* < 0.05) and the protein expression of β-Catenin (*P* < 0.05) in IL-1β + P group was significantly increased compared with that in IL-1β group.

## Discussion

To the best of our knowledge, this is the first study to investigate the effect of PEMF on subchondral bone degeneration at the early stage of TMJOA and explored its possible molecular mechanism.

OA is one of the most common joint disorders worldwide [29]. Mechanical stresses on TMJ that originated from daily functional activity such as talking, chewing, etc., are thought to be significant for remodeling of the mandibular condyles [30]. Joint tissues are exquisitely sensitive to their mechanical environment, and mechanical loading may be the most important external factor regulating the development and long-term maintenance of joint tissues [31]. Aberrant biomechanical stimulation from abnormal dental occlusion plays an important role in TMJOA process [32]. In the present study, diffuse subchondral bone loss, enlargement of bone marrow cavity, rough and uneven surface of subchondral bone were observed in UAC group, accompanied with decreased BV/TV and Tb. Th, and increased BS/BV and Tb. Sp, especially at 6 W time-point after modeling, which were consistent with our previous study [32]. Therefore, it is an effective animal model to evaluate the effect of different intervention factors on the prognosis of the early subchondral bone resorption during TMJOA process.

It is well known that abnormal subchondral bone remodeling plays an important role in the pathogenesis of OA, and abnormal condylar subchondral bone absorption is the main pathological characteristics in the early stages of TMJOA. As for the management of this kind of bone absorption, intra-articular injection of hyaluronic acid (HA) and platelet-rich plasma (PRP) may be a promising way without regard to its invasiveness [33]. As a non-invasive and safe physical therapy method, PEMF has been proven to promote fresh/delayed fracture healing and inhibit bone loss caused by disuse or estrogen deficiency with no observable side effects [34]. To examine the effects of PEMF therapy on symptoms and quality of life in patients with OA, a meta-analysis on 15 clinical studies was conducted by Yang XT et al. in 2020, which concluded that there was a beneficial effect of PEMF therapy on pain, stiffness, and physical function in patients with OA [35]. Appropriate PEMF exposure (75 Hz, 1.6 mT, 2 h/d, 4 W) can preserve the structural integrity of subchondral bone in rats knee OA induced by low-dose monosodium iodoacetate (MIA) injection by activating OPG/RANKL/RANK signaling [19]. In addition, appropriate PEMF exposure (8 Hz, 3.8 mT, 40 min/d, 4 d) can substantially reduce the number of osteoclast-like cells in the culture with M-CSF + RANKL, and subsequent bone resorption, at least partially, through NFATc1, CAII and RANK [36]. However, it is still unknown whether appropriate PEMF exposure could effectively reverse condylar subchondral bone loss during TMJOA process. In the present study, the abnormal bone resorption, the increased number of osteoclasts, the decreased expression of osteogenesis-related factors, the increased expression of osteoclast-related factors and OPG/RANKL ratio induced by UAC, were reversed by PEMF exposure (2mT, 15 Hz, 2 h/d, 3 or 6 W) to the normal level similar to that of CON group. It is suggested that abnormal subchondral bone remodeling at the early stage of TMJOA could also be reversed effectively by PEMF, similar to other large joints of limbs, although TMJ is different from them in anatomy. The present study offers an exciting new avenue to interfere with the early progress of TMJOA.

It is well known that estrogen play an important role in maintaining bone homeostasis, and estrogen supplement may be beneficial to patients with early OA or postmenopausal osteoporotic osteoarthritis [37]. In the present study, similar to the effect of PEMF exposure, estrogen supplement can also reverse the abnormal bone resorption, the increased number of osteoclasts, the decreased expression of osteogenesis-related factors, the increased expression of osteoclast-related factors and OPG/RANKL ratio induced by UAC, which was consistent with our previous study [27]. It is indicated that the effect of PEMF exposure is similar to that of high physiological concentration of estrogen. Unfortunately, estrogen replacement therapy is reported to be associated with a higher risk for breast, endometrial and ovarian cancer as well as cardiovascular disease and stroke [38]. Taken this side effect into consideration, appropriate PEMF exposure is a better way to reverse abnormal subchondral bone remodeling at the early stage of TMJOA. Interestingly, in the present study, the combination of E_2_ supplement and PEMF exposure not only reversed the effect of estrogen on the body weight of rats at the early stage of TMJOA, but also further increased the ratio of OPG/RANKL, the expression of some osteogenesis-related factors and Tp. Sp of micro-CT at 6 W. It is suggested that E_2_ supplement and PEMF exposure may have a synergistic effect to some extent, which can further improve the osteogenic potential of subchondral bone in TMJOA.

Considerable evidence supports the crucial role of canonical Wnt signaling pathway (β-Catenin-dependent) in regulating bone mass and architecture and maintaining bone homeostasis [20, 39]. Wnt/β-Catenin signal promotes osteogenic activity by stimulating the proliferation and differentiation of osteoblasts [40]. In 2016, Zhai M et al. found that PEMF exposure (2 mT, 15.38 Hz, 2 h/day) can increase expressions of ALP and OCN at the differentiation phase, then upregulated expressions of COL1A1, Runx2 and Wnt/β-Catenin signaling (Wnt1, LRP6, and β-Catenin) at proliferation and differentiation phases in osteoblasts [22]. Similarly, Fathi E et al. found that ZnSO4, in the presence of PEMF exposure (20mT, 50 Hz, 30 min/day, 21 days), resulted in an increase in the expression of osteogenic genes (OCN, Runx2 and BMP2), ALP activity and calcium levels during osteogenic differentiation of adipose tissue-derived mesenchymal stem cells (ADSCs), accompanied with increased expressions of β-Catenin, Wnt1, Wnt3a, LRP5 and DKK1 [41]. In the present study in vivo and in vitro, we found that PEMF can also up-regulate Wnt/β-Catenin signal pathway, promoting the entry of β-Catenin into the nucleus and inhibiting GSK3β activity. It is suggested that Wnt/β-Catenin signal pathway is invovled in the effect of PEMF exposure in reversing abnormal subchondral bone remodeling at the early stage of TMJOA.

The present study has several limitations. Firstly, although PEMF intervention reversed the abnormal resorption of subchondral bone significantly in rats, the optional PEMF parameters and duration for the treatment of human TMJOA should still be investigated carefully in the future. Additionally, except the Wnt/β-Catenin signal pathway, several other signal pathways may be involved in the effect of PEMF intervention on osteogenesis, for example, mitogen-activated protein kinase (MAPK) pathway, Ca^2+^ ionic pathway, etc. Which pathway plays the most important role in the process deserves further investigation.

## Conclusion

In this study, we have demonstrated that PEMF intervention can reverse the abnormal resorption of subchondral bone by promoting the expression of osteogenesis-related factors and inhibiting the expression of osteogenesis-related factors at the early stage of TMJOA induced by UAC, which is similar to the effect of estrogen. In addition, PEMF exposure and estrogen supplement may have a synergistic effect to some extent, which can further improve the osteogenic potential of subchondral bone in TMJOA. Furthermore, the preservation of bone formation in TMJOA is associated with the activation of Wnt/β-Catenin signal pathway induced by PEMF. This study provides direct evidence that PEMF stimulation, as an easy and non-invasive biophysical technique, has promising therapeutic potential at the early stage of TMJOA.

## Supplementary Information

**Additional file 1.**

## Data Availability

All data generated or analysed during this study are included in this published article.
