# Correction: Low frequency pulsed electromagnetic fields exposure alleviate the abnormal subchondral bone remodeling at the early stage of temporomandibular joint osteoarthritis

**DOI:** 10.1186/s12891-022-06113-y

**Published:** 2023-01-19

**Authors:** Yuanjun Ma, Xiaohua Chen, Feng He, Shi Li, Rui He, Qian Liu, Qingshan Dong, Shuncheng Zhou, Hui Miao, Qian Lu, Feifei Li, Hongxu Yang, Mian Zhang, Yuan Lin, Shibin Yu

**Affiliations:** 1 State Key Laboratory of Military Stomatology, National Clinical Research Center for Oral Diseases, Shaanxi Key Laboratory of Oral Diseases, School of Stomatology, the Fourth Military Medical University, Xi’an, Shaanxi 710032 People’s Republic of China; 2 Department of Stomatology, Chinese PLA General Hospital of Central Theater Command, Wuhan, 430070 People’s Republic of China; 3 Department of Stomatology, Seventh Medical Center of Chinese PLA General Hospital, Beijing, 100700 People’s Republic of China

**Correction: BMC Musculoskelet Disord 23, 987 (2022)**

10.1186/s12891-022-05916-3

Following publication of the original article [1], the authors reported errors in the second and third columns of Fig. 7. The result of RANKL mRNA expression appears to be identical to OCN mRNA expression (2nd column); and the result of the OCN mRNA expression appears to be identical to the ALP mRNA expression (3rd column). Errors were introduced when the author provided replacement Fig. 7 due to figure's low quality.

**Fig. 7:**
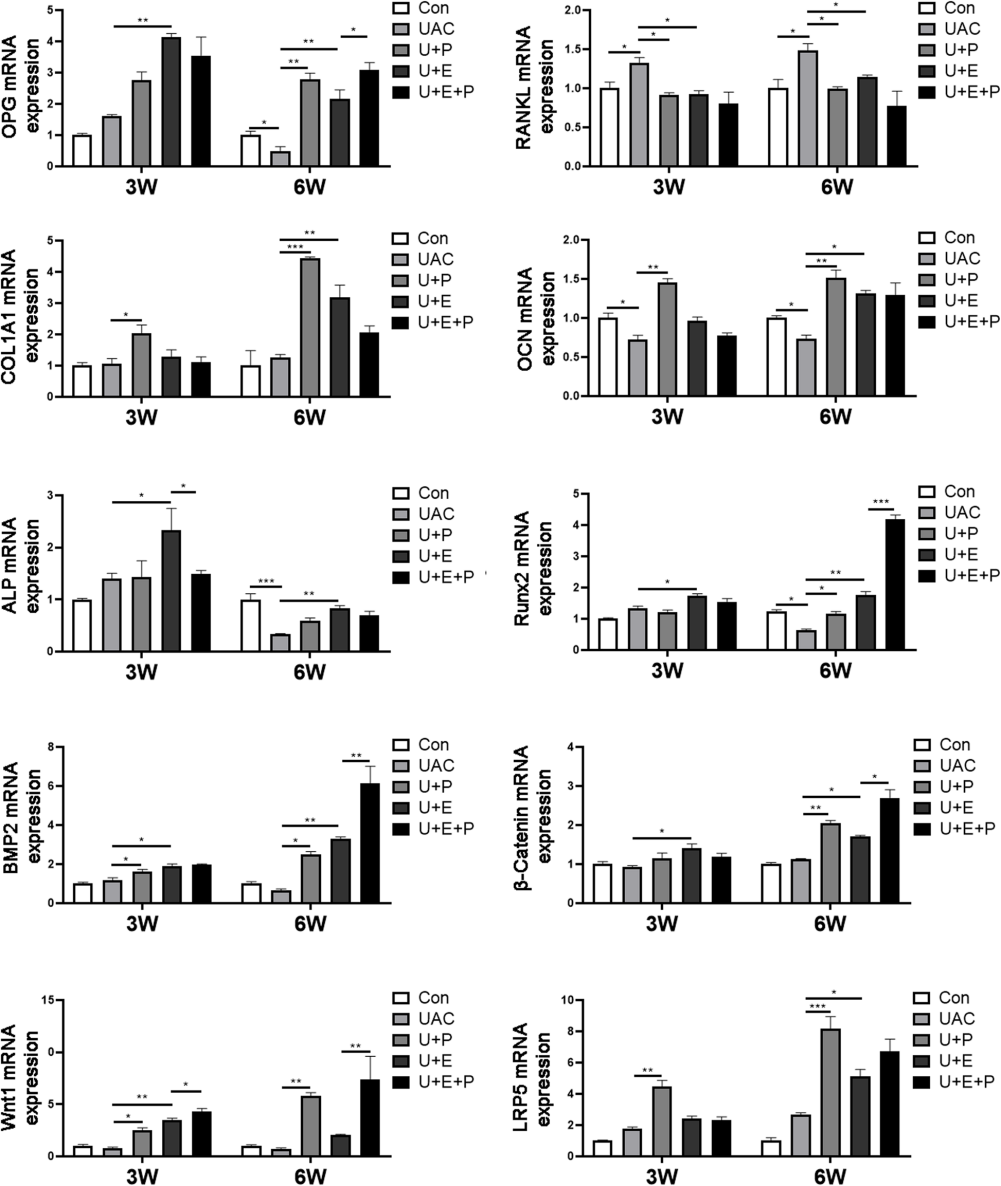
The mRNA expression of osteogenesis-related factors (*n*=6). *: *P*< 0.05, **: *P*< 0.01, ***: *P*< 0.001

The original article [1] has been updated.

Below is the corrected Fig. 7.
