# Supplementary material for: Low frequency pulsed electromagnetic fields exposure alleviate the abnormal subchondral bone remodeling at the early stage of temporomandibular joint osteoarthritis
Source: BMC Musculoskelet Disord. 2022 Nov 16;23:987. doi: 10.1186/s12891-022-05916-3 (PMC9667650; doi:10.1186/s12891-022-05916-3)
Supplement: Supplementary file 1 — Additional file 1. [file 12891_2022_5916_MOESM1_ESM.pdf]

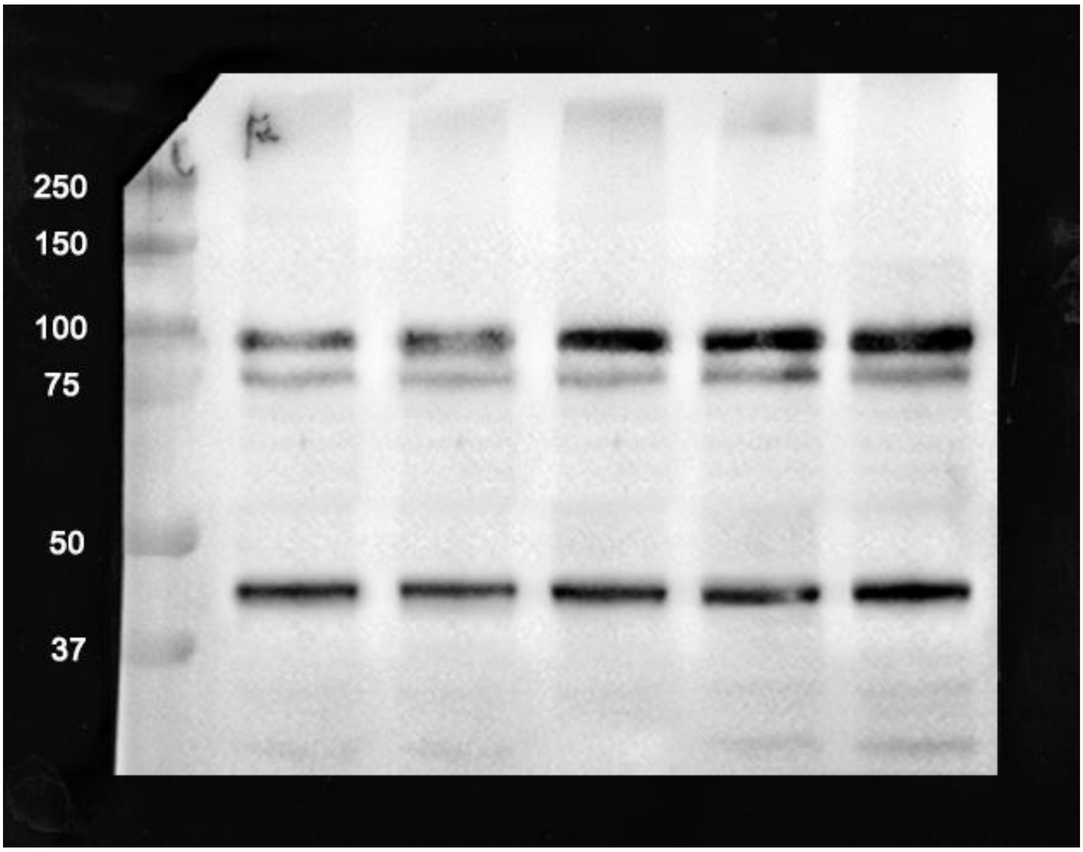

Originals of fig 6: β-Catenin (92 kD) and GSK3β (46 kD) at 3 W

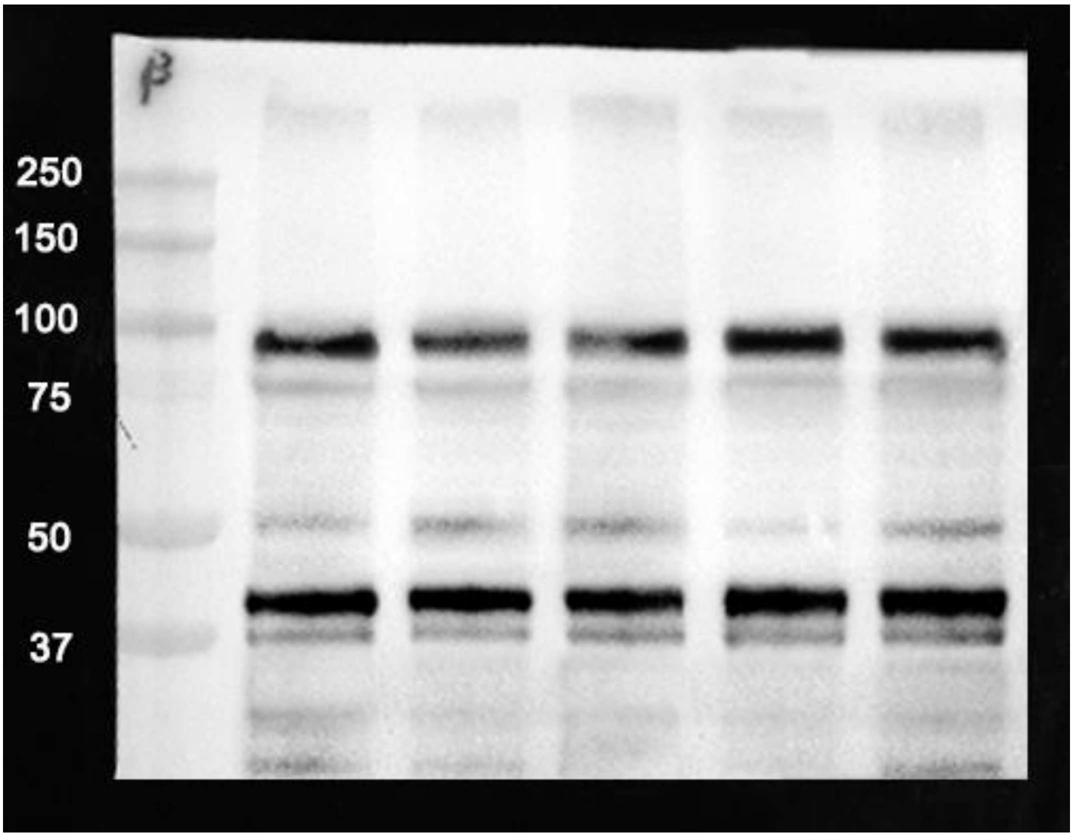

Originals of fig 6: β-Catenin (92 kD) and GSK3β (46 kD) at 6 W

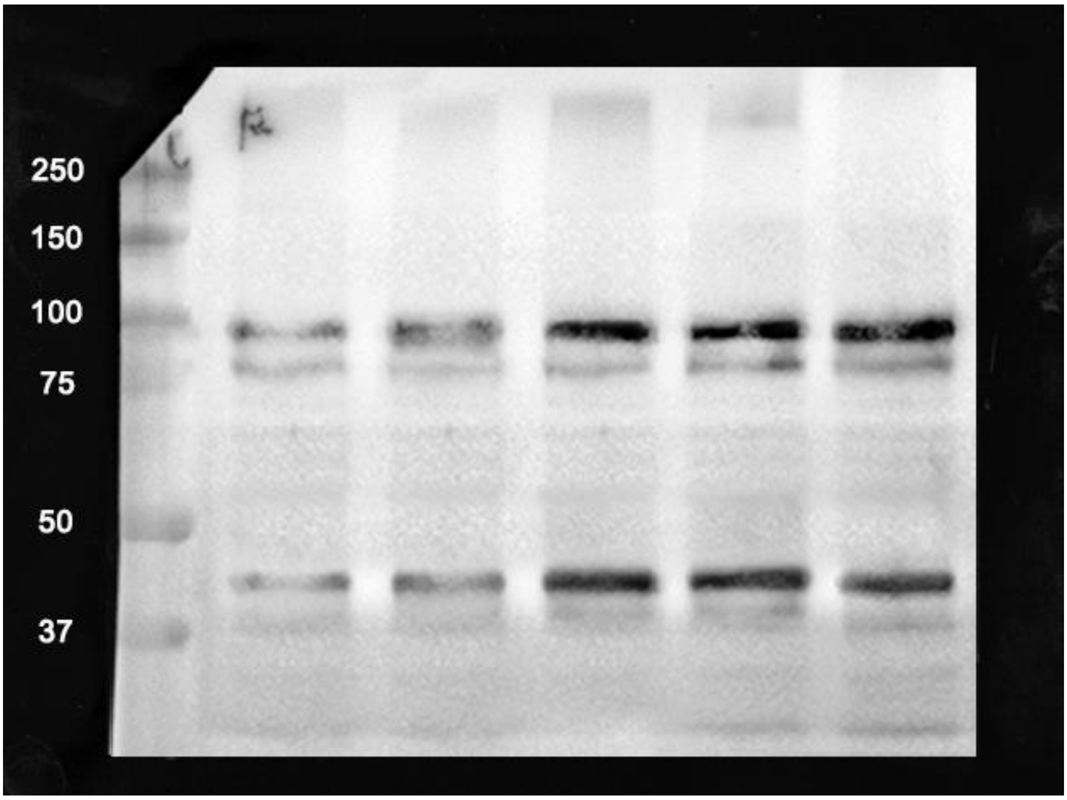

Originals of fig 6: OPG (46 kD) at 3 W

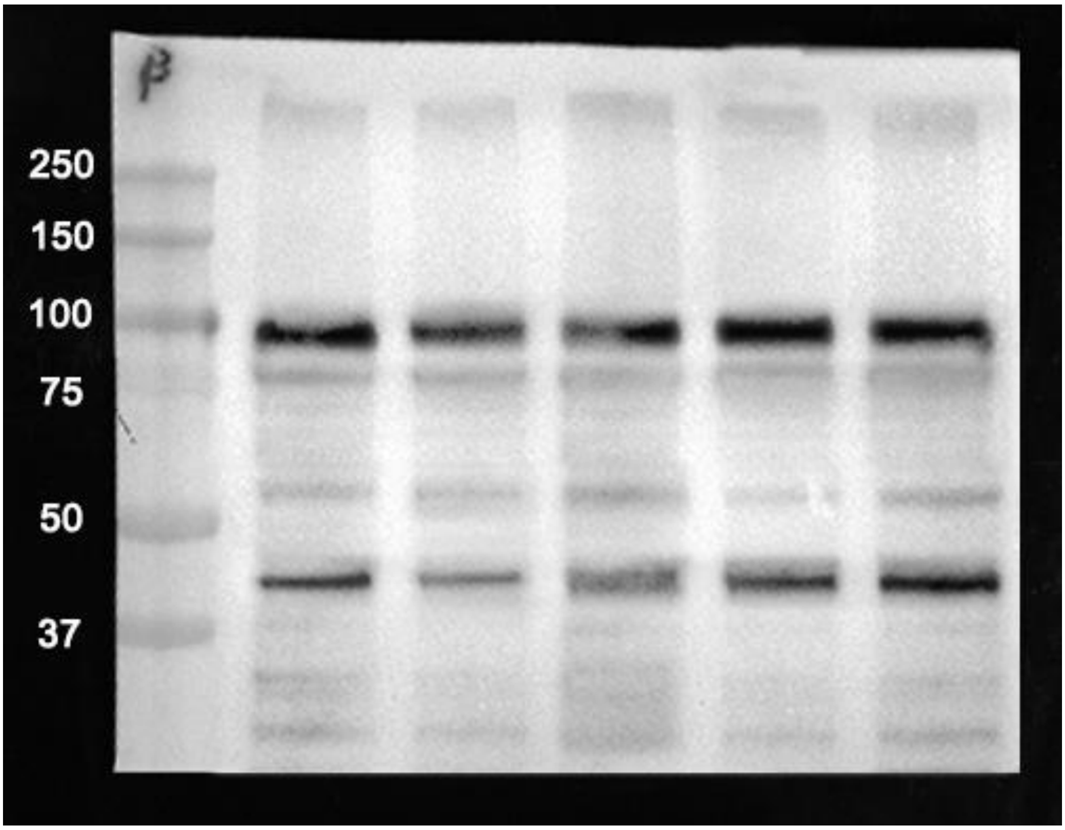

Originals of fig 6: OPG (46 kD) at 6 W

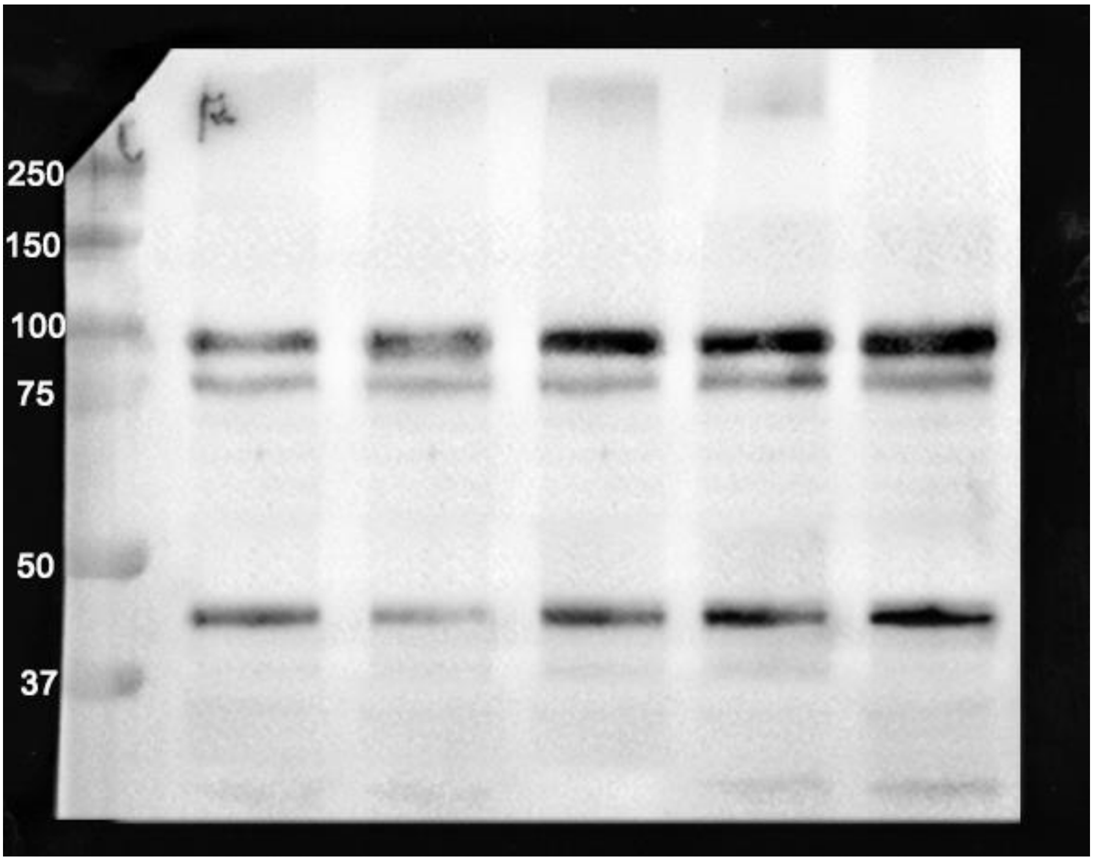

Originals of fig 6: p-GSK3β (46 kD) at 3 W

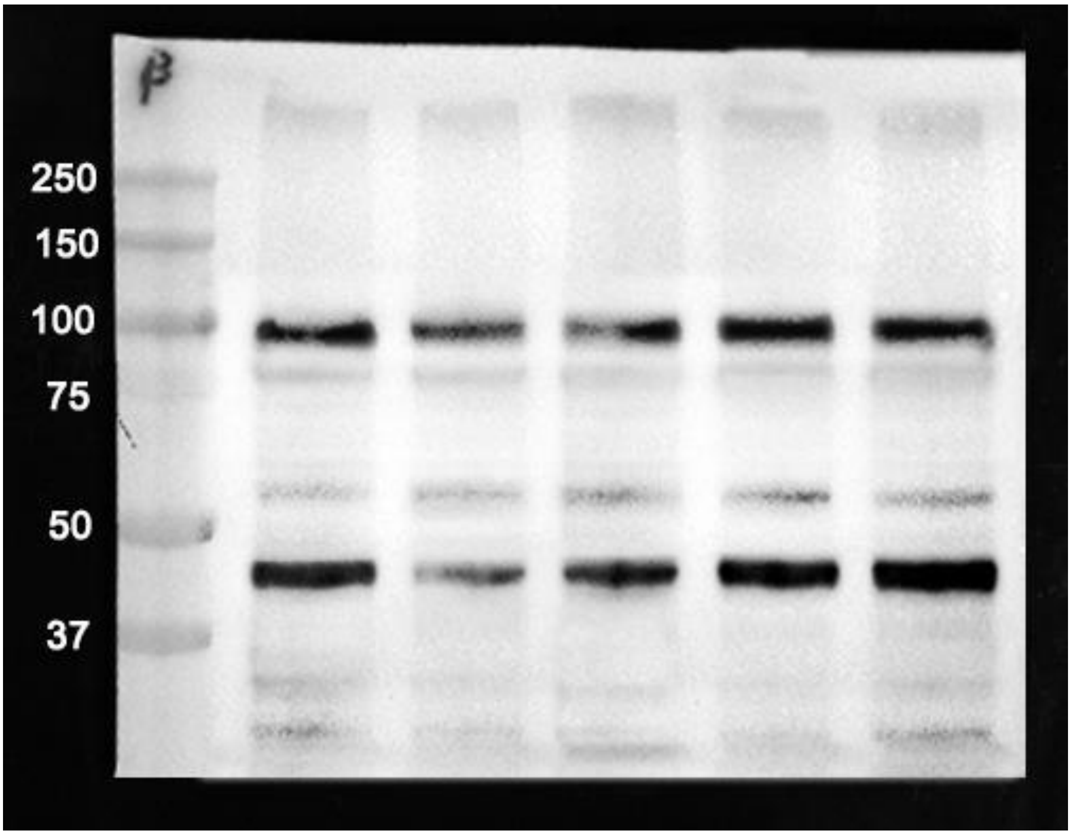

Originals of fig 6: p-GSK3β (46 kD) at 6 W

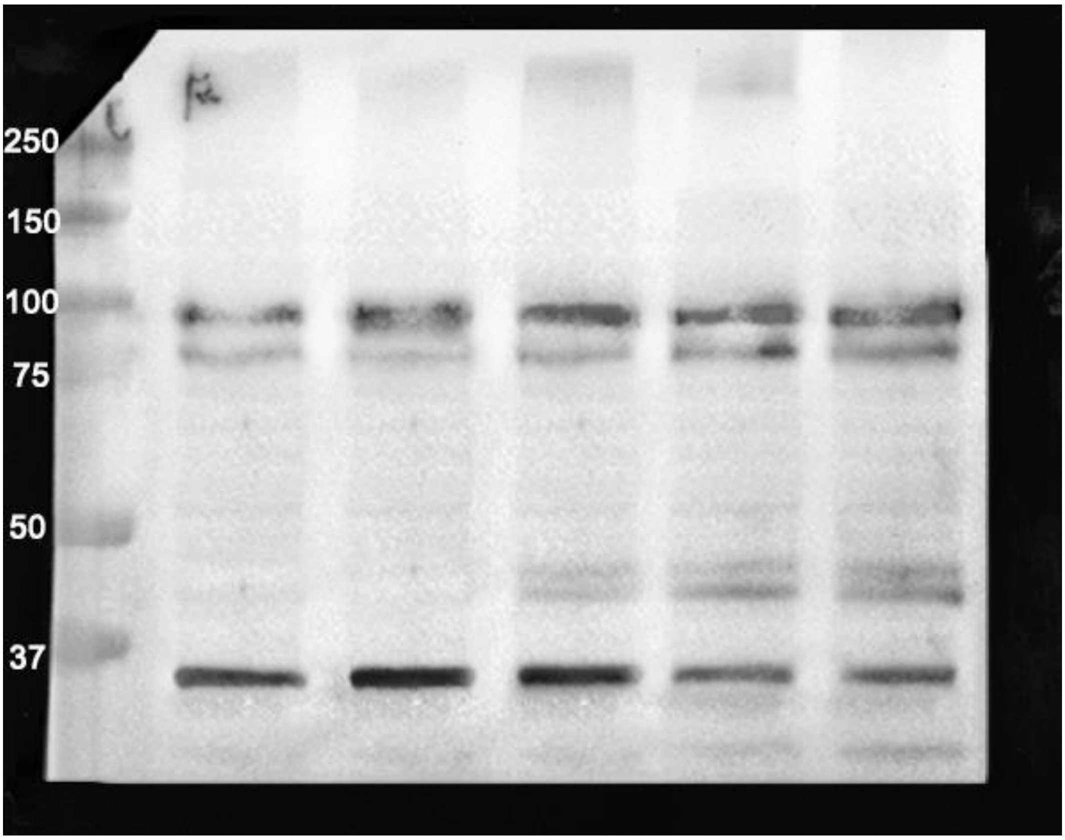

Originals of fig 6: RANKL (35 kD) at 3 W

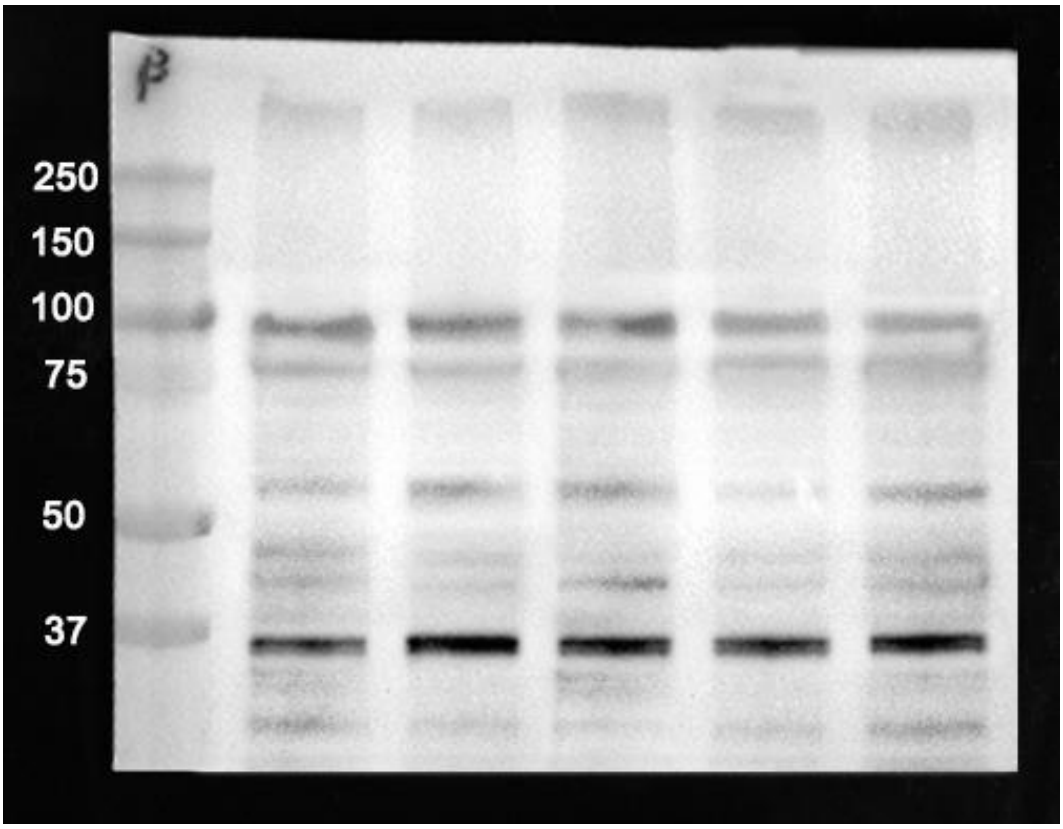

Originals of fig 6: RANKL (35 kD) at 6 W

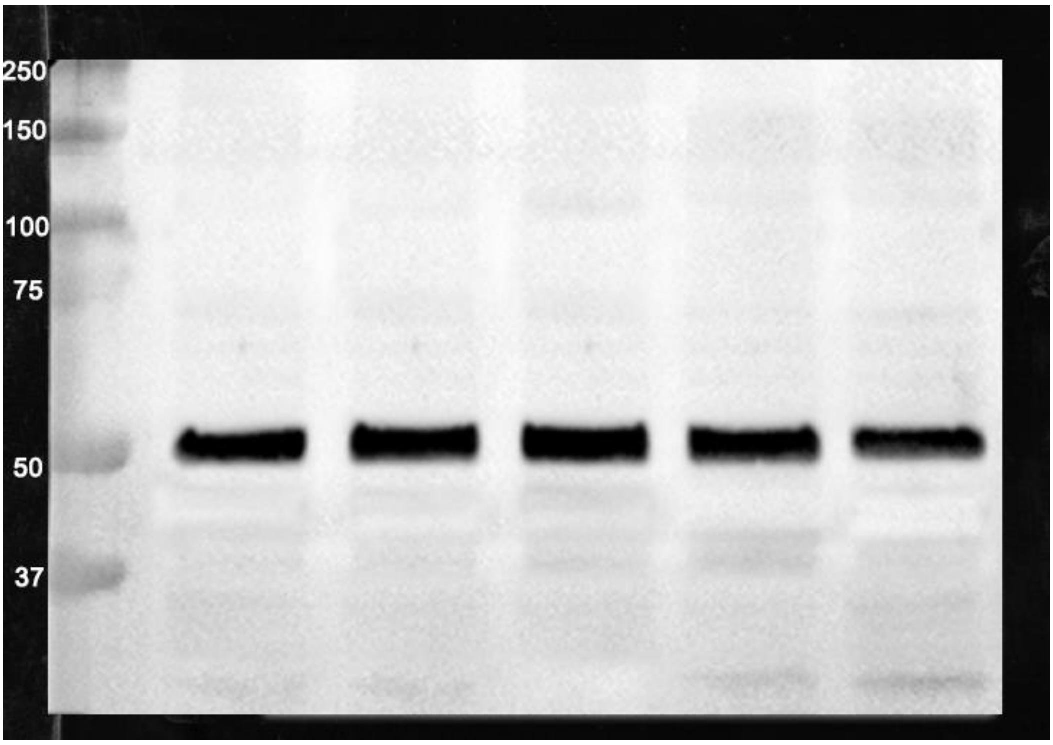

Originals of fig 6: β-Tubulin (50 kD) at 3 W

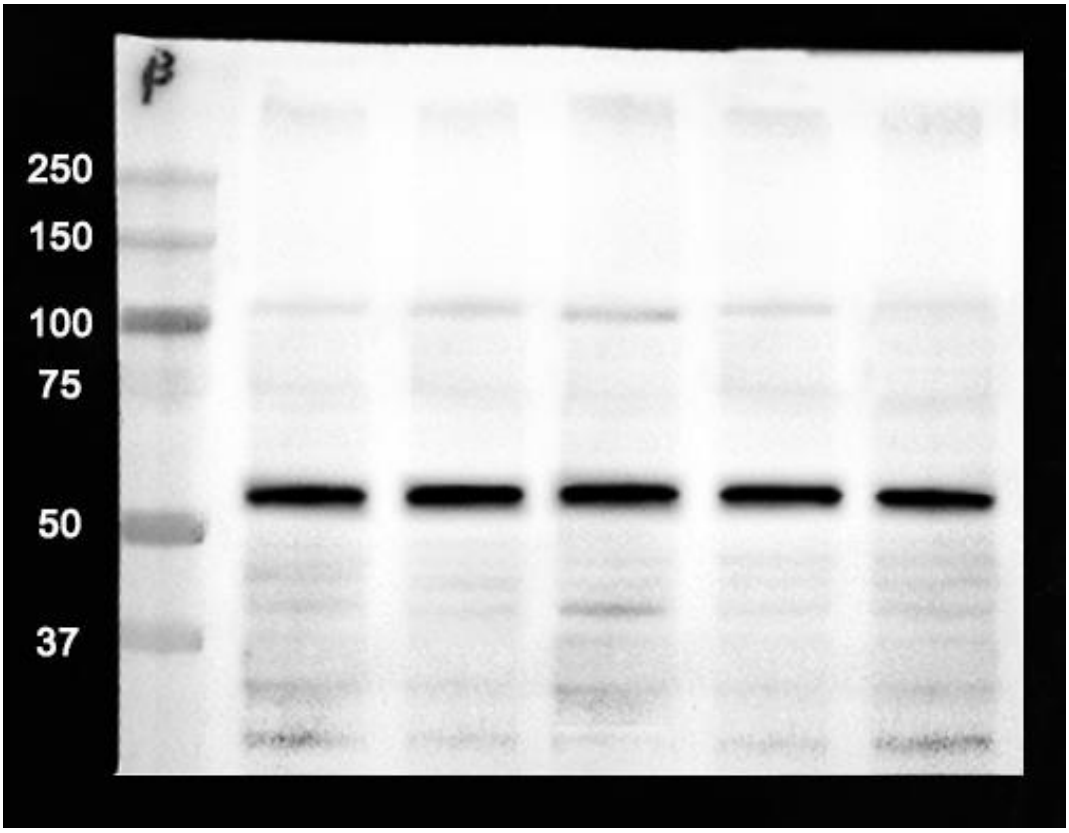

Originals of fig 6: β-Tubulin (50 kD) at 6 W

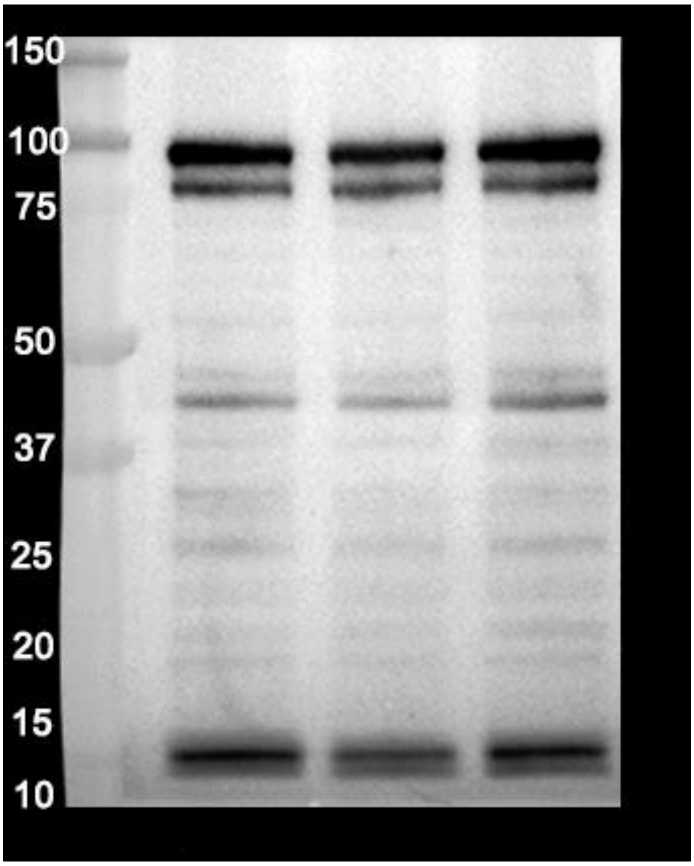

Originals of fig 7: β-Catenin (92 kD) and OCN (11 kD)

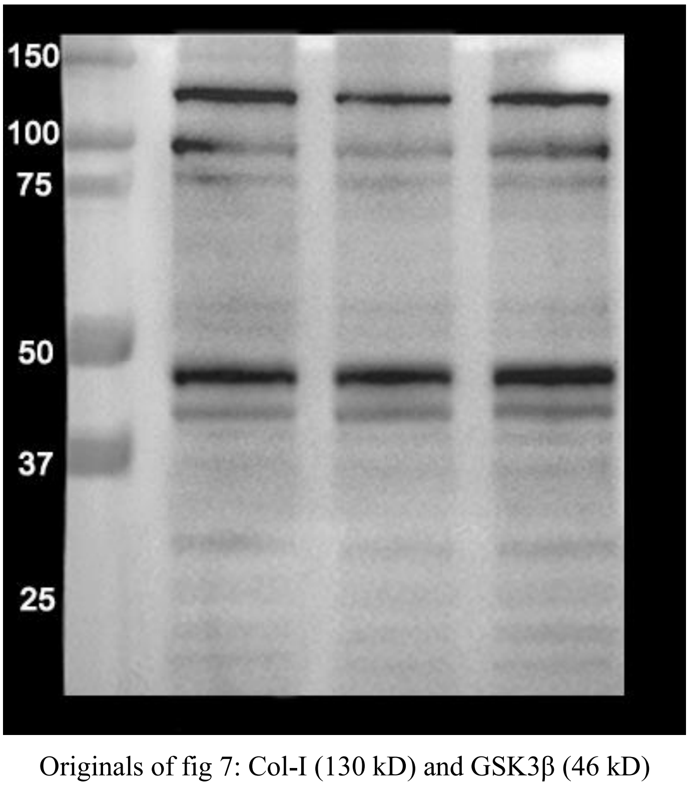

Originals of fig 7: Col-I (130 kD) and GSK3β (46 kD)

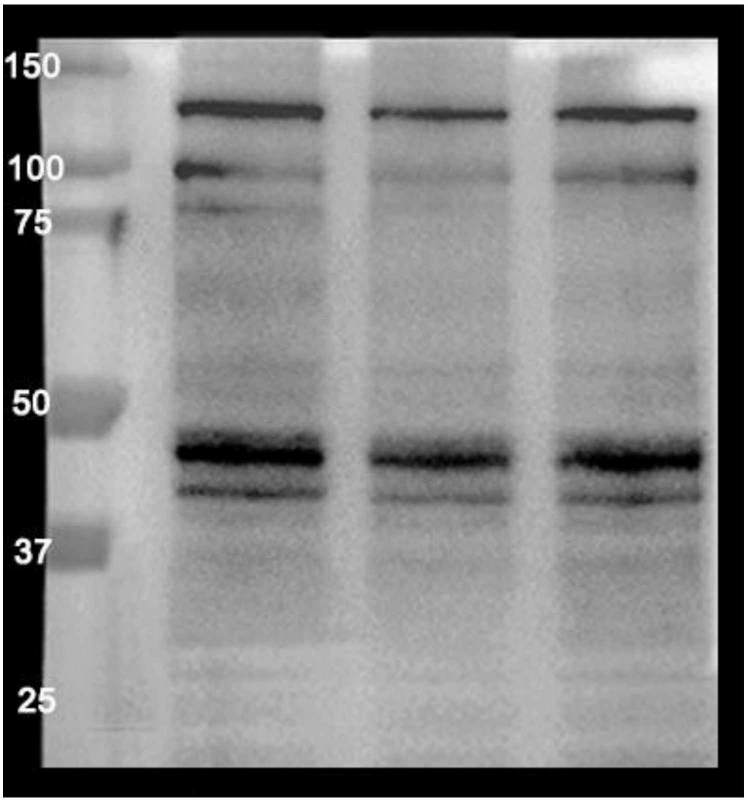

Originals of fig 7: p-GSK3β (46 kD)

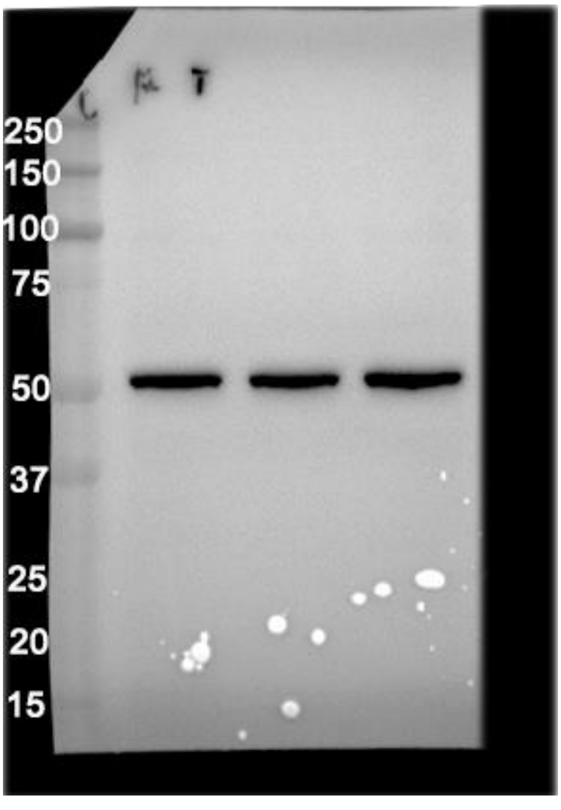

Originals of fig 7: β-Tubulin (50 kD)
